# Can G-quadruplex become a promising target in HBV therapy?

**DOI:** 10.3389/fimmu.2022.1091873

**Published:** 2022-12-15

**Authors:** Ye Teng, Ming Zhu, Yuan Chi, Lijing Li, Ye Jin

**Affiliations:** ^1^ School of Pharmacy, Changchun University of Chinese Medicine, Changchun, China; ^2^ Pharmaceutical Department, The Affiliated Hospital of Changchun University of Chinese Medicine, Changchun, China

**Keywords:** G-quadruplex, chronic hepatitis B virus, interferon-stimulated gene, antiviral therapy, gene expression

## Abstract

The chronic infection with hepatitis B virus (HBV) is an important health problem that affects millions of people worldwide. Current therapies for HBV always suffer from a poor response rate, common side effects, and the need for lifelong treatment. Novel therapeutic targets are expected. Interestingly, non-canonical structures of nucleic acids play crucial roles in the regulation of gene expression. Especially the formation of G-quadruplexes (G4s) in G-rich strands has been demonstrated to affect many bioprocesses including replication, transcription, and translation, showing great potential as targets in anticancer and antiviral therapies. In this review, we summarize recent antiviral studies about G4s and discuss the potential roles of G4 structures in antiviral therapy for HBV.

## Introduction

Chronic hepatitis B virus (HBV) infection remains a global health problem that impacts more than 250 million people (1). HBV infection can further progress to complications such as cirrhosis and hepatocellular carcinoma (HCC), which cause more than 780,000 deaths annually (2). Effective treatment of HBV is urgently needed to significantly reduce the progression of liver disease. Currently, there are two kinds of drugs for the treatment of HBV infection: interferons (IFNs) and nucleoside/nucleotide analogs (NAs). IFNs are a group of cytokines released by host cells to protect them from viral infection (3, 4), and they modulate the immune response against viruses by stimulating the expression of IFN-stimulated genes (ISGs). IFN therapy can inhibit viral replication, degrade viral components, and induce T-cell growth (5). However, only one third of the patients achieve a sustained response, and the undesirable side effects of IFN therapy are common, including influenza-like illness, fatigue, weight loss, anorexia, and emotional lability (2, 6). On the other hand, NAs directly target the reverse transcription of pregenomic mRNA to DNA and inhibit the replication of HBV. But the change in envelope protein might induce immune escape (7), and the patients must face drug resistance during the indefinite treatment. Though many studies of the combination therapy of IFNs and NAs were conducted in recent years, there is still a lack of evidence on the effectiveness (8–11). A novel target for HBV therapy is required to improve current treatment.

**Figure 1:**
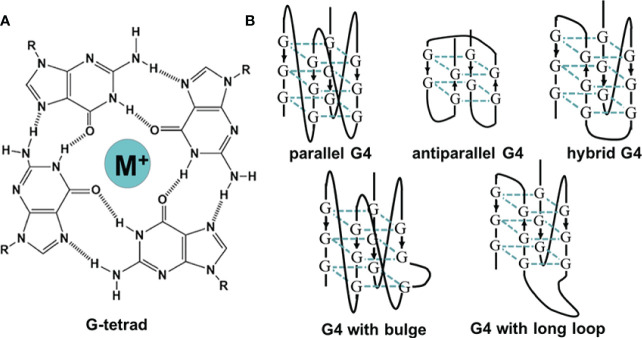
**(A)** The illustration of G-tetrad. **(B)** The schematic of G-quadruplexes with different topologies.

**Figure 2:**
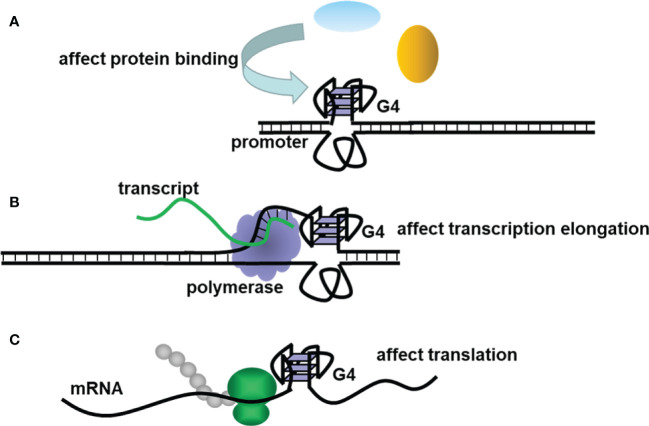
The schematic diagram of G4s regulating **(A)** promoter activity, **(B)** transcription, and **(C)** translation.

Non-canonical structures of nucleic acids are now becoming promising targets in the research of various diseases. The secondary structures of nucleic acids are important for their function. Unlike double-helix DNAs, which are used to store genetic information, non-canonical structures, such as triplex, G4, and i-motif, are involved in the regulation of many bioprocesses (12). G4 is a typical non-canonical structure composed of π–π stacked G-tetrads, in which four guanines interact with each other through Hoogsteen base pairing (**Figure 1A**). G4-forming sequences are widely found in the human genome (13). Researchers have observed the presence of both DNA and RNA G4s in cells utilizing fluorescent G4-specific antibodies, and they could be targeted through G4 binding ligands (14, 15). G4s have various topological structures, which can be classified into parallel, antiparallel, and hybrid structures by the orientation of their strands (**Figure 1B**). Moreover, non-canonical G4 structures such as G4s with long loops or bulges are also commonly present, which might provide more binding sites for nucleotides, proteins, and ligands (13). The topology and stability of G4 are determined by not only its sequence but also the surrounding environment (16). For example, the presence of cations such as K^+^ could significantly stabilize G4s (17). Under the molecular crowding conditions, the excluded volume effects of cosolutes, water activity, and the dielectric property of the solution are also crucial for G4s (18). The stability changes lead to topology changes or the folding or unwinding of G4s, thus affecting the interaction between G4s and proteins and ultimately realizing their regulatory roles in gene expression. Correspondingly, in the analysis of the human genome, a great number of G4s are in regulatory regions such as promoters and untranslated regions, participating in multiple biological processes, including replication, transcription, and translation (19–22). **Figure 2** presents the typical examples of G4s regulating gene expression, including the alteration of the interaction between promoter and functional proteins by G4s in the promoter region, the inhibition of transcription by G4s in the coding region, and the inhibition of translation by G4s in mRNAs (12).

Based on these characteristics, G4s have shown great potential in the research of many diseases. For example, G4s exist in many cancer-associated genes like Myc (23). They are important targets in anticancer studies due to their multiple functions, including the inhibition of oncogene expression, the suppression of telomerase activity, the selectivity for targeting cancer cells, and so on (24, 25). G4s have also been demonstrated to play key roles in viral infection, altering viral replication, transcription, and translation (26). On the other hand, G4-binding ligands attract great interest owing to their special binding affinity. The interaction between G4 and its ligand could significantly enhance or reduce the effect of G4 on biological processes, indicating the potential of G4s as a novel therapeutic target (27). In the past decades, a variety of G4 ligands have been developed through artificial synthesis or natural extracts. Li’s group has built a database for G4 ligands since 2013 (28), and more than 3,000 kinds of G4 ligands were included with predicted ligand binding affinity and real-time ligand–receptor docking, providing a comprehensive tool for the discovery of drug candidates. Some G4-binding ligands such as CX-5461 have achieved significant development in clinical trials for safety, tolerability, and pharmacokinetics (29), further explaining the potential applications of G4s in future therapies.

The antiviral effects of G4s make them promising targets for HBV-related studies (26). They might directly regulate viral gene expression, and the different therapeutic mechanism from IFN suggest the possibility of combination treatment. In this review, recent antiviral research on G4s was summarized, and the alternation of HBV gene expression was introduced in detail. The potential roles of G4s in HBV infection and treatment were discussed to provide a new perspective.

## G4s as antiviral targets

G4s are promising antiviral targets due to their widespread existence in a variety of viruses and their regulatory function in gene expression. The study of G4s in viruses has started for decades since an intermolecular G4 structure was found in human immunodeficiency virus (HIV) genomic RNA in 1993 (30). Up to now, G4s have been widely found in the genomes of various viruses, including HIV, herpes simplex virus (HSV), hepatitis C virus (HCV), human papillomavirus (HPV), Zika virus (ZIKV), Epstein–Barr virus (EBV), Kaposi’s sarcoma-associated herpesvirus (KSHV), severe acute respiratory syndrome coronavirus (SARS-CoV), human cytomegalovirus (HCMV), and so on (31). **Table 1** lists examples of G4-forming sequences found in different viruses and their secondary structures, and the majority of G4s display parallel structures. Moreover, Lavezzo et al. searched for potential G4-forming sequences in the genomes of all known human viruses with a computational method (57). The potential G4 sequences were searched in patterns of (G_2_N_1–7_)_3_G_2_, (G_3_N_1–12_)_3_G_3_ and (G_4_N_1–12_)_3_G_4_, and the results suggested that G4s were widely existing, and were more than expected from the composition. Double-stranded RNA and DNA viruses had a relative lack of G-rich islands compared with single-stranded viruses. It provided us with a comprehensive perspective on G4 presence in the viral study. With the development of genomics and bioinformatics, the existence of more G4s has been confirmed, and their biological functions have attracted the attention of researchers. The locations and functions of G4s in viral genomes are summarized in **Table 2**, and the interactions between G4 and G4 ligands/proteins were also involved.

**Table 1:** Examples of G4 forming sequences with structural characterization in different viral genomes.

Virus	DNA/RNA	G4 forming sequences (5’-3’)	G4 structures	References
HIV-1	DNA	CTGGGCGGGACTGGGGAGTGGT	Parallel G4 with a T bulge	(32)
	DNA	TGGCCTGGGCGGGACTGGG	Antiparallel G4	(33)
	DNA	GGGGACTTTCCAGGGAGGCGTGGCCTGGGCGGG	Parallel G4 with long loops	(34)
	DNA	GGGAGGCGTGGCCTGGGCGGGACTGGGG	Hybrid G4 with a stem-loop	(35)
	DNA	GGAGGAGGAGGTGGG	Parallel G4	(36)
	DNA	GGGGGGACTGGAAGGG	Parallel G4	(36)
	RNA	AGG GAG GCG UGG CCU GGG CGG GAC UGG GGA GUG GC	Parallel G4	(37)
HCV	RNA	GGGCUGCGGGUGGGCGGGA	Parallel G4	(38, 39)
	RNA	GGGCAUGGGGUGGGCAGGA	Parallel G4	(38)
	RNA	GGGCGAAGGUCCUGGUGG	Parallel G4	(39)
	RNA	GGUGGAGGGUGAGG	Parallel G4	(39)
	RNA	GGGGGGGUGGGUGG	Parallel G4	(39)
	RNA	GGCGGAGGAGG	Parallel G4	(39)
	RNA	GGAGGUUAAGGCAGCGG	Parallel G4	(39)
ZIKV	RNA	GUGGAAGAGUGAUAGGACUCUAUGGCAAUGGGGUU	Parallel G4	(40)
	RNA	GUGGAGGUGGGACGGGAG	Parallel G4	(40)
	RNA	UCGGAUGUGGCAGAGGGGGCUGGAG	Parallel G4	(40)
	RNA	GCGGCGGCCGGUGUGGGGAA	Parallel G4	(40)
	RNA	GGAGUGGGAAGCGG AGCUGG	Parallel G4	(41)
	RNA	GGACCGCCUGGGGG UGGGGGGAGG	Parallel G4	(41)
	RNA	GGUGGGGGACUGG	Parallel G4	(41)
	RNA	GGCAUGGGGGAGG	Parallel G4	(41)
SARS-CoV-2	RNA	UGGCUGGCAAUGGCGGU	Parallel G4	(42)
	RNA	UGGAGGAGGUGUUGCAGGA	Parallel G4	(42)
	RNA	GGUAUGUGGAAAGGUUAUGG	Parallel G4	(43)
	RNA	GGCUUAUAGGUUUAAUGGUAUUGG	Parallel G4	(43)
	RNA	GGAUAUGGUUGGUUUGG	Parallel G4	(44)
HSV-1	DNA	GGGGCTGGGGCTGGGGTTGGGG	Antiparallel G4	(45, 46)
	DNA	GGGGGCGAGGGGCGGGAGGGGGCGAGGGG	Antiparallel G4	(45, 46)
	DNA	GGGAGGAGCGGGGGGAGGAGCGGG	Parallel G4	(45, 46)
	DNA	GGGGGAGAGGGGAGAGGGGGGGAGAGGGG	Parallel G4	(46)
EBV	DNA	GGGGGGGGTAGGGGGGGG	Parallel G4	(47)
	RNA	AGGAGGUGGAGG	Hybrid G4	(48)
	RNA	AGGAGCAGGAGGUGG	Hybrid G4	(48)
	RNA	GGGGCAGGAGCAGGAGGA	Parallel G4	(49)
HCMV	DNA	GGGCCGGGACGGGGTGGG	Parallel G4	(50)
	DNA	GGGGTGGGAGGGACTTTTGCGGGTAGTGCATGCTAAGATGAACGGGTGGGCTGGGG	Parallel G4	(51)
	DNA	GGGGCACCCGGGTGTGGCGCTACGGG	Antiparallel G4	(51)
	DNA	GTGGTGTGGGGCCCGTGAGGGGGAGTCGTTGGG	Hybrid G4	(51)
KSHV	DNA	GGGGTGTGGGATGGGGGTGTGGG	Hybrid G4	(47)
	DNA	GGGTGGGAGGAGGAAGGATGTGGGGGTGGG	Parallel G4	(50)
	DNA	GGGGCGGGGGACGGGGGAGGGG	Parallel G4	(52)
	DNA	GGGGCTCGGGGCTCGGGGCCCCGGGG	Parallel G4	(52)
HPV	DNA	GTGGGAGCGGGAACGGGAACGGGA	Parallel G4	(53)
	DNA	AGTGGTACAGGGGGTCGTACAGGGTACATTCCATTGGGTGGGCG	Parallel G4	(53)
	DNA	GGGAGTATGGGTAACGGGGGGGG	Parallel G4	(53)
	DNA	GGGAAAGGGTACCTCGAGGGGCCGCGGGG	Parallel G4	(53)
	DNA	GGGTAGGGCAGGGGACACAGGGT	Hybrid G4	(53)
	DNA	GGGCAGGGTAGGGCAATTTAGGG	Hybrid G4	(53)
HBV	DNA	GGGAGTGGGAGCATTCGGGCCAGGG	Hybrid G4	(54)
	DNA	CTGGGAGGAGCTGGGGGAGGAGA	Parallel G4	(55)
	DNA	GGCTGGGGCTTGGTCATGGGCCATCAG	Hybrid G4	(56)

**Table 2:** Overview of locations, functions of G4s in different viruses, and typical G4 binding ligands and proteins used in related studies.

Virus	Viral genome	Locations of G4s	Processes/Functions affected by G4s	Typical G4 binding ligands	Typical G4 interacting proteins	References
HIV-1	ssRNA (RT)	LTR promoter, 5’ end of the gag gene, nef coding region	Promoter activity, transcription, nef protein expression, HIV-1 infectivity	BRACO-19, TMPyP4, c-exNDI	nucleolin, hnRNP A2/B1, HIV-1 nucleocapsid protein	(34, 36, 37, 58–62)
HCV	ssRNA (+)	HCV core gene, 3’ end of negative strand	Replication, translation	PDP, TMPyP4, PDS, PhenDC3	Nucleolin, HCV nonstructural protein NS3	(38, 39, 63, 64)
ZIKV	ssRNA (+)	Coding regions for prM, E, NS1, NS2, NS3, NS4B, and NS5 proteins, 3’-UTR	Replication, viral protein synthesis	TMPyP4, BRACO-19, PDS		(40, 41, 65, 66)
SARS-CoV-2	ssRNA (+)	5’ UTR, ORF1 ab, spike, ORF3a, membrane, and nucleocapsid genes	Viral life cycle, nonstructural proteins expression	TMPyP4, PhenDC3, BRACO-19, PDS, CX-3543, berberine	Nucleolin, CNBP, hnRNPs, RNA helicases, viral helicase nsp13	(42–44, 67–74)
HSV-1	dsDNA	Inverted repeats, immediate early promoter	Replication, transcription, viral production, recombination	BRACO-19, TMPyP4, c-exNDI		(45, 46, 75–78)
EBV	dsDNA	mRNA of EBNA1, BHRF1 promoter	EBNA1 mRNA translation, viral Bcl-2 expression	BRACO-19, PDS, PyDH2, PhenDH2, PhenDC3	Nucleolin	(47, 49, 79–81)
HCMV	dsDNA	Promoter regions including unique long, unique short, terminal and internal repeat regions, promoter of miR-US33	Viral gene expression, miRNA promoter activity	TMPyP4, NMM, CX-5461, PDS		(50, 51, 82)
KSHV	dsDNA	Terminal repeat region, LANA1 mRNA, promoter of KS-Bcl-2, promoters of miR-K12–1-9,11	Viral replication, LANA expression, viral Bcl-2 expression, miRNA promoter activity	PhenDC3, PhenDH2 TMPyP4, PDS		(47, 48, 50, 52, 83)
HPV	dsDNA	Long control region, coding region for L1, L2, E1, and E4	Viral protein production, viral gene expression	TMPyP4, BRACO-19, PDS, 360A, PhenDC3, C8		(53, 84–86)
HBV	dsDNA (RT)	PreS2/S promoter, coding region of polymerase protein, pre-core promoter of cccDNA	Promoter activity, protein production	TMPyP4, BRACO-19, PDS	DHX36	(54–56)

G4s exist in both the noncoding and coding regions of viruses. Their functions are dependent on their locations. Promoter is one of the most common locations of G4s, suggested the importance of G4s in gene regulation. For example, researchers found G4s in the HIV-1 long terminal repeat (LTR) promoter, and the G-rich region overlapped with NF-κB and SP1 binding sites, showing crucial effects on promoter activity (34). Moreover, both DNA and RNA G4s were found in the U3 region of HIV-1, and multiple intermolecular G4s might form to affect the template switching of reverse transcription (37). G4s with long loops and bugles in the immediate early promoters of HSV 1 and 2 and varicella zoster virus (VZV) were identified to tune the promoter activity at the cellular level (75). In addition, G4s were found in other noncoding regions, such as the 3’-UTR in the ZIKV genome, which was essential for viral replication (40). G4s also widely existed in the coding regions of viral genes, which directly affected their transcription and replication. For example, there are reports describing the presence of G4s in nef, tat, rev, env, and vpx coding regions in the HIV-1 gene (36, 87) and coding regions of prM, E, NS1, NS2, NS3, NS4B, and NS5 proteins in the ZIKV genome (40, 41). Many of them are important virulence factors, which are crucial for us to understand the effects of G4s in the viral life cycle and pathogenesis. Moreover, G4s in EBNA1 mRNA of EBV and LANA1 mRNA of KSHV were characterized to reveal the multiple functions of RNA G4s, such as the disruption of the interaction with the origin recognition complex and the control of translation to achieve immune recognition restriction (48, 49). The widespread existence and diverse function of G4 make it an important factor in viral gene expression.

Usually, G4 acts as a switch in the regulation of gene expression. The stabilization of G4s could significantly decrease the activity of the promoter and silence viral gene expression, while the unwinding of G4s could enhance the promoter activity and resume higher efficiency in viral transcription and replication (84, 88). Therefore, G4s are considered promising antiviral targets, and the study of G4 binding ligands has been one of the most attractive areas for years. **Table 2** lists typical G4-binding ligands reported in recent antiviral research. BRACO19, TMPyP4, PhenDC3, and PDS are most used G4 ligands, which could significantly stabilize G4 formation and show antiviral activity. For example, the presence of BRACO19 was demonstrated to stabilize G4s in the LTR promoter, decrease its activity, and repress the transcription of HIV-1 (58). BRACO19 could also combine with G4s in the genome of EBV to suppress viral replication (89). G4 ligands and their biological effects in antiviral studies have been reviewed in detail, revealing the potential of G4 ligands in the development of novel antiviral therapies *via* G4-mediated pathways (27, 31, 90). However, some G4 ligands had relatively poor selectivity between G4s and duplexes or other quadruplexes, DNA G4s and RNA G4s, and different G4 structures, which hindered the clinical application of G4 ligands (27). Although there are still some problems with their application, G4 ligands do exhibit excellent antiviral activity. Further understanding of their mechanism would expand the pharmacological application of G4 ligands.

G4-binding proteins are also the focus of research because they are involved in replication, transcription, and mRNA processing (91). They could be divided into two groups, proteins stabilized G4s, and proteins unfolded G4s. Nucleolin is the most studied protein to stabilize G4s in antiviral research. For example, it was demonstrated to bind G4s in the LTR promoter and silence HIV-1 transcription (59). Nucleolin was also found to bind to HCV core RNA G4s and suppress HCV replication (39). On the other hand, proteins that can unwind G4s are essential for the replication and repair of viral genes. Viral-encoded proteins, such as HIV-1 nucleocapsid protein NCp7, HCV nonstructural protein NS3, and SARS-CoV nonstructural protein 13 (nsp13), were demonstrated to efficiently unfold G4s to allow effective replication and enhanced transcription (60, 63). Interestingly, the unfolding of G4s by these proteins could be reduced in the presence of G4 binding ligands such as PDS and BRACO-19, suggesting the potential of G4s and their ligands in antiviral therapies (60, 63). G4-binding proteins in host cells could also be utilized by viruses in their replication, which are considered better targets to achieve a wide response using common cellular pathways. For example, human nuclear ribonucleoprotein A2/B1 (hnRNP A2/B1) efficiently unfolds G4s to enhance HIV-1 transcription (61). Cellular nucleic acid binding protein (CNBP) was also demonstrated to interact with G4s in the SARS-CoV-2 RNA genome and promote their unfolding (44). These studies provide us with an advanced understanding of viral replication and transcription mechanisms and show the great potential of G4-targeted ligands and proteins in antiviral treatment.

In addition, making artificial G4s in the viral genome to inhibit viral gene expression is another application of G4s in antiviral research. For example, stable G4s have been demonstrated to block viral transcription and replication. A strategy using a tetraethylene glycol-modified oligonucleotide that could form an intermolecular G4 with the GG sequence in HIV-1 was designed to block the reverse transcription of HIV-1 and suggested a promising therapeutic method utilizing the function of G4 (92).

## Roles of G4s in HBV genome

**Figure 3:**
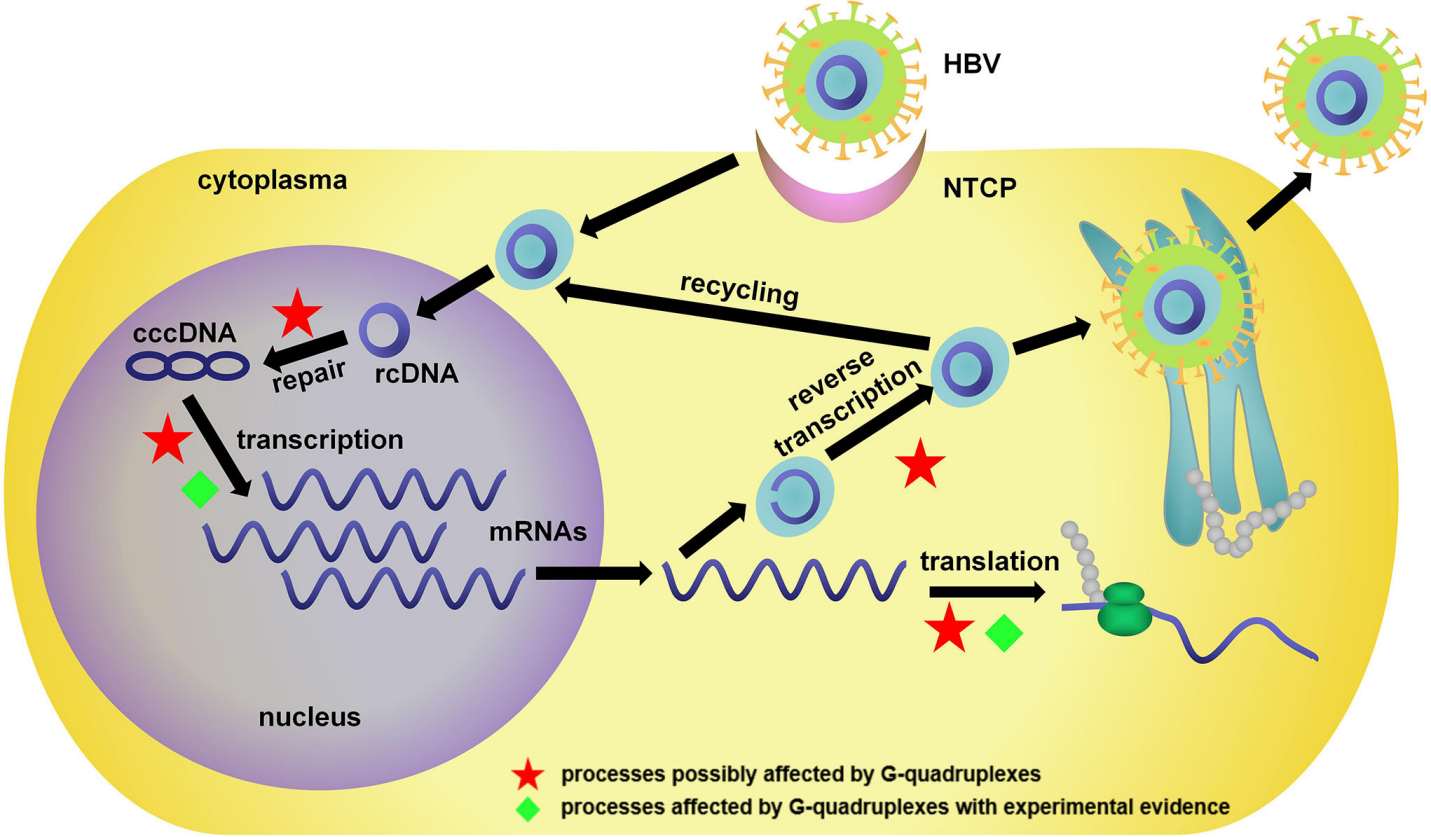
Life cycle of HBV and related bioprocesses possibly affected by G4s. NTCP, sodium taurocholate cotransporting polypeptide; rcDNA, relaxed circular DNA; cccDNA, covalently closed circular DNA.

HBV is a partially double-stranded DNA virus with a relaxed circular DNA (rcDNA) genome of approximately 3.2 kb (93). As shown in **Figure 3**, in HBV infection, the virus interacts with its receptor, the bile acid transporter NTCP, and enters the cell. The rcDNA is then released into the nucleus and converted into covalently closed circular DNA (cccDNA). The transcribed pregenomic mRNA further translates to core proteins and polymerase and serves as the template for reverse transcription to rcDNA. The newly formed rcDNAs are enveloped and then released as new virions or recycled for cccDNA repair.

The confirmation of detailed G4 in HBV is relatively late compared with HIV. The first evidence of G4 formation in the HBV genome was reported in 2017. Biswas and coworkers analyzed full-length HBV sequences with the pattern G_3–4_N_1–7_ and found a G4-forming sequence in the presS2/S promoter of HBV genotype B (54). The structure was further confirmed as a hybrid intramolecular G4 by circular dichroism (CD), nuclear magnetic resonance, native polyacrylamide gel electrophoresis, and DMS footprinting analysis. The formation of G4 in the preS2/S promoter was related to promoter activity, and mutations in G4 resulted in the reduction of preS2/S transcripts and HBsAg levels. As HBsAg was the major surface protein in the HBV envelope, virion secretion was greatly affected by the presence of G4.

Meier-Stephenson and Badmalia discovered another unique G4 in the pre-core promoter of HBV cccDNA, which was the central part of pregenomic RNA generation and protein synthesis (55). A 23-mer oligomer in wild-type promoter region and three mutant types with a single-nucleotide mutation of G to A were characterized by CD and small-angle X-ray scattering. Parallel G4 structures were observed in both the wild and mutant types. Interestingly, the combination of G4 and the G4 binding protein DHX36 significantly pulled down HBV cccDNA. Moreover, the HepG2 transfection study demonstrated that, compared with the mutant ones, the expression of HBsAg and HBeAg was significantly decreased in the wild type. In contrast, the level of HBcAg was increased, suggesting the formation of G4 had different effects on viral core protein production.

Molnár analyzed the effects of cations and G4 ligands on a G4 structure in the HBV genome (56). A hybrid G4 was observed in the presence of K^+^, while a parallel structure was induced in the presence of Na^+^, Li^+^, and Rb^+^. The interaction between G4 ligands TMPyP4, BRACO19, and PhenDC3 was characterized in detail as well. This provided more information about the possible roles of G4 and its ligands in the fight against HBV.

As a double-stranded DNA virus, HBV has a relatively low level of G4-forming sequences compared with single-stranded DNA/RNA viruses (57). Notably, the limited G4s in key regions could still be a crucial factor in the regulation of gene expression. The red stars in **Figure 3** indicate processes possibly affected by G4s. These works provided sufficient evidence for the existence of G4s in the HBV genome, and as novel antiviral targets, their potential functions were investigated in HBV infection.

## Roles of G4s in immune genes

**Figure 4:**
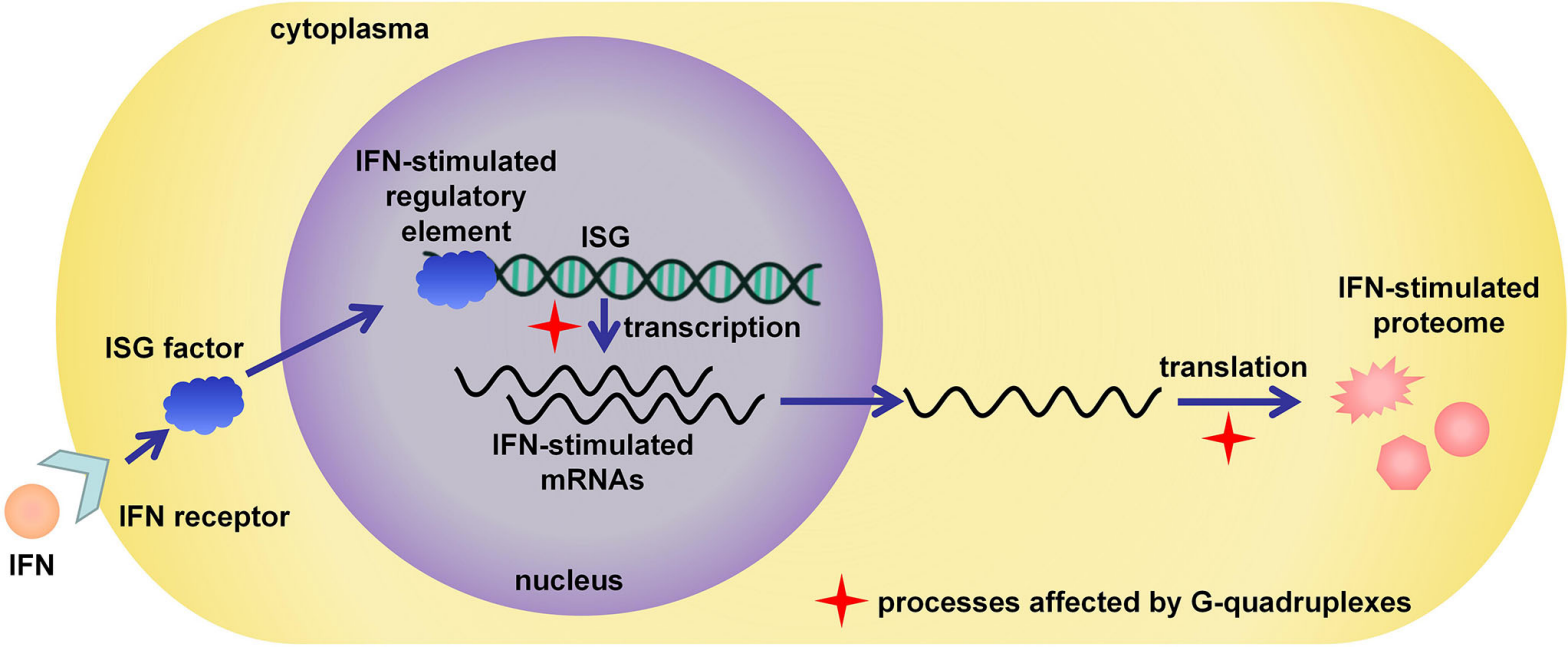
IFN-stimulated antiviral response and related bioprocesses possibly affected by G4s.

Targeting G4s in related host genes is also a significant strategy in antiviral studies. Immune response is important in virus infection; therefore, ISGs are considered promising targets for antiviral therapy (94, 95). For example, IFNs released by host cells could stimulate the expression of ISGs to significantly suppress the infection and replication of the virus; therefore, IFNs have been approved for use in the clinical treatment of HBV infection (2, 6). Recent research has also revealed that the structure of nucleic acid is a critical factor involved in the immune response. For example, the long noncoding RNA lnc-MxA was reported to form a DNA/RNA triplex and inhibit the transcription of IFN-β (96). G4, as an important functional non-B structure, showed great potential to affect the innate immune system and the expression of ISGs. The IFN-stimulated gene expression was shown schematically in **Figure 4**, and the red cross stars indicated the processes affected by G4s. The Seimiya group found that the formation of G4 in telomere repeats inhibited the expression of innate immune genes, including STAT1, ISG15, and OAS3 (97). Later, they reported that G4s bind to splicing factor 3B subunit 2 (SF3B2), suppress STAT1 expression and ISG induction (98). The induction occurred with various G4-forming sequences and could not be observed in the presence of non-G4 sequences, even though they had the same lengths or guanine contents as G4-forming sequences. Moreover, the presence of the G4 ligand Phen-DC3 blocked the interaction between SF3B2 and G4s and reversed the inhibition of ISG induction. It demonstrated that G4s were important for ISG expression and might be promising therapeutic targets in antiviral studies.

The effects of G4 on ISG were also investigated in HBV infection. Sun reported a novel mechanism of STAT1 involved IFN signaling based on RNA structural change (99). Based on the work of Kwok on RNA G4 sequencing in the human transcriptome (100), a G-rich sequence at the 5’ UTR in STAT1 mRNA was found to form RNA G4 and participate in the regulation of STAT1 expression. Importantly, DDX5, an RNA helicase involved in transcription, RNA splicing, and translation, was demonstrated to selectively bind to the G4 structure in STAT1 mRNA and promote the translation of STAT1 protein. Notably, in the IFN treatment of HBV infection, the knockdown of DDX5 decreased the level of STAT1 protein because of G4 formation in mRNA, and resulted in the reduction of IFN-α antiviral effect. This research discussed how the RNA structural changes affected IFN response, providing us a novel insight into the mechanism of IFN treatment.

## Discussion

Non-canonical structures of DNAs and RNAs are promising targets in antiviral therapy, especially G4s. The presence of G4s has been demonstrated in various viral genomes to regulate their expression. In HBV studies, targeting G4s could regulate the development of HBV infection in two ways. One is targeting G4s located in the promoter regions of the HBV genome, which can regulate viral replication and transcription. Mutations that disrupt the formation of G4s could significantly decrease the expression level. G4-binding proteins and G4 ligands are promised to control the viral life cycle by blocking the interaction between G4 and host proteins in the initial stages of replication and transcription. The other one is targeting G4s in ISG genes. The presence of G4s could inhibit the transcription of ISGs, thus affecting the therapeutic effect of IFN treatment. The fact that the repression of ISGs could be reversed by G4-binding proteins and ligands suggested their potential in the modulation of immune response. With the development of more G4s in HBV and related ISGs, our understanding of their function in the viral life cycle and HBV treatment will improve.

In addition, G4s are involved in other functional RNAs, such as miRNAs and lncRNAs (101–103). Notably, both miRNAs and lncRNAs play key roles in HBV infection and HBV-related HCC, suggesting G4s and their ligands could affect the progress of HBV and HBV-related HCC through novel routes (104–107). For example, metastasis-associated lung adenocarcinoma transcript 1 (MALAT1) is a non-coding RNA that regulates alternative splicing in HBV-related HCC (104). Three parallel G4s were recently found in the 3’ region of MALAT1, which interacted with nucleolin and affected the alternative splicing of RNA transcripts (103). Similar results were also observed in miRNA studies. The formation of G4 in miRNA was demonstrated to affect miRNA-mRNA interaction and regulate miRNA-mediated post-transcription (108). The G4s in functional RNAs might provide a new perspective for us in the development of novel targets in HBV therapy.

However, there are still some challenges to overcome before targeting G4s in the clinical treatment of HBV infection. First, most of the thermodynamic analysis of G4s was conducted *in vitro*. As the stability of G4 is greatly affected by its surrounding environment, whether *in vitro* experiments accurately mimic the internal environment of infected cells is a problem. Notably, analytical methods have been built to achieve the visualization of G4s and their structural transition utilizing G4 antibodies or fluorescent probes in recent years (14, 109). More valuable information about the structural change and regulation mechanism of G4s in cells is expected in the future. Second, the selectivity of G4 ligands might be a potential risk factor. There has been evidence showing that some G4 ligands could bind to other non-canonical structures like the i-motif (110). It was suggested that the mechanism of G4 ligand-induced effects might be complicated. Third, it is difficult for G4 ligands to bind with a specific G4. This means they might target multiple G4s in different locations in the genome at the same time. In the comparison of the interactions between different G4s and typical ligands, including TMPyP4, BRACO19, PDS, PhenDC3, and TrisQ, none of them showed obvious selectivity for a specific topology (111). Now researchers are trying to improve the selectivity of G4 ligands to avoid unexpected side effects or cellular toxicity (112). Moreover, more clinical data is necessary for antiviral application. Now the clinical trials of G4 ligands are mainly about anticancer therapy, and some of them have achieved significant progress (29). G4s and their binding proteins/ligands could directly affect the expression of viral genes; therefore, as promising targets, G4s should not be ignored in HBV therapy. Regardless, G4-related research provides a special perspective on HBV treatment, and novel strategies targeting the regulation of G4s are expected in the future.

## Author contributions

All authors listed have made a substantial, direct, and intellectual contribution to the work, and approved it for publication.

## References

[1] Razavi-Shearer D Gamkrelidze I Nguyen MH Chen D-S Van Damme P Abbas Z. Global prevalence, treatment, and prevention of hepatitis b virus infection in 2016: A modelling study. Lancet Gastroenterol Hepatol (2018) 3(6):383–403. doi: 10.1016/S2468-1253(18)30056-6 29599078

[2] Ye J Chen J. Interferon and hepatitis b: Current and future perspectives. Front Immunol (2021) 12:733364. doi: 10.3389/fimmu.2021.733364 34557195 PMC8452902

[3] Xiang Y Guo Z Zhu P Chen J Huang Y. Traditional Chinese medicine as a cancer treatment: Modern perspectives of ancient but advanced science. Cancer Med (2019) 8(5):1958–75. doi: 10.1002/cam4.2108 PMC6536969 30945475

[4] Dunn GP Koebel CM Schreiber RD. Interferons, immunity and cancer immunoediting. Nat Rev Immunol (2006) 6(11):836–48. doi: 10.1038/nri1961 17063185

[5] Buster EHCJ Schalm SW Janssen HLA. Peginterferon for the treatment of chronic hepatitis b in the era of Nucleos(T)Ide analogues. Best Pract Res Clin Gastroenterol (2008) 22(6):1093–108. doi: 10.1016/j.bpg.2008.11.007 19187869

[6] Konerman MA Lok AS. Interferon treatment for hepatitis b. Clin Liver Dis (2016) 20(4):645–65. doi: 10.1016/j.cld.2016.06.002 27742005

[7] Chan HL-Y Thompson A Martinot-Peignoux M Piratvisuth T Cornberg M Brunetto MR. Hepatitis b surface antigen quantification: Why and how to use it in 2011 – a core group report. J Hepatol (2011) 55(5):1121–31. doi: 10.1016/j.jhep.2011.06.006 21718667

[8] Janssen HLA van Zonneveld M Senturk H Zeuzem S Akarca US Cakaloglu Y. Pegylated interferon Alfa-2b alone or in combination with lamivudine for hbeag-positive chronic hepatitis b: A randomised trial. Lancet (2005) 365(9454):123–9. doi: 10.1016/S0140-6736(05)17701-0 15639293

[9] Lau GKK Piratvisuth T Luo KX Marcellin P Thongsawat S Cooksley G. Peginterferon Alfa-2a, lamivudine, and the combination for hbeag-positive chronic hepatitis b. N Engl J Med (2005) 352(26):2682–95. doi: 10.1056/NEJMoa043470 15987917

[10] Cao ZH Ma LN Zhang HW Liu YL Chen XY. Extended treatment with peginterferon A-2a in combination with lamivudine or adefovir for 96 weeks yields high rates of hbeag and hbsag seroconversion. J Dig Dis (2013) 14(8):446–50. doi: 10.1111/1751-2980.12065 23615131

[11] Wong GL Wong VW Chan HL. Combination therapy of interferon and Nucleotide/Nucleoside analogues for chronic hepatitis b. J Viral Hepat (2014) 21(12):825–34. doi: 10.1111/jvh.12341 25402543

[12] Tateishi-Karimata H Sugimoto N. Roles of non-canonical structures of nucleic acids in cancer and neurodegenerative diseases. Nucleic Acids Res (2021) 49(14):7839–55. doi: 10.1093/nar/gkab580 PMC8373145 34244785

[13] Chambers VS Marsico G Boutell JM Di Antonio M Smith GP Balasubramanian S. High-throughput sequencing of DNA G-quadruplex structures in the human genome. Nat Biotech (2015) 33(8):877–81. doi: 10.1038/nbt.3295 26192317

[14] Biffi G Tannahill D McCafferty J Balasubramanian S. Quantitative visualization of DNA G-quadruplex structures in human cells. Nat Chem (2013) 5(3):182–6. doi: 10.1038/nchem.1548 PMC3622242 23422559

[15] Biffi G Antonio M Tannahill D Balasubramanian S. Visualization and selective chemical targeting of rna G-quadruplex structures in the cytoplasm of human cells. Nat Chem (2014) 6:75–80. doi: 10.1038/nchem.1805 24345950 PMC4081541

[16] Fujii T Podbevšek P Plavec J Sugimoto N. Effects of metal ions and cosolutes on G-quadruplex topology. J Inorg Biochem (2017) 166:190–8. doi: 10.1016/j.jinorgbio.2016.09.001 27665315

[17] Wang Y Patel DJ. Guanine residues in D(T2ag3) and D(T2g4) form parallel-stranded potassium cation stabilized G-quadruplexes with anti glycosidic torsion angles in solution. Biochemistry (1992) 31(35):8112–9. doi: 10.1021/bi00150a002 1525153

[18] Nakano S Miyoshi D Sugimoto N. Effects of molecular crowding on the structures, interactions, and functions of nucleic acids. Chem Rev (Washington DC U S) (2014) 114(5):2733–58. doi: 10.1021/cr400113m 24364729

[19] Hänsel-Hertsch R Di Antonio M Balasubramanian S. DNA G-Quadruplexes in the human genome: Detection, functions and therapeutic potential. Nat Rev Mol Cell Biol (2017) 18:279. doi: 10.1038/nrm.2017.3 28225080

[20] Tateishi-Karimata H Isono N Sugimoto N. New insights into transcription fidelity: Thermal stability of non-canonical structures in template DNA regulates transcriptional arrest, pause, and slippage. PloS One (2014) 9(3):e90580. doi: 10.1371/journal.pone.0090580 24594642 PMC3940900

[21] Endoh T Kawasaki Y Sugimoto N. Translational halt during elongation caused by G-quadruplex formed by mrna. Methods (2013) 64(1):73–8. doi: 10.1016/j.ymeth.2013.05.026 23747335

[22] Teng Y Tateishi-Karimata H Sugimoto N. C-rich sequence in a non-template DNA strand regulates structure change of G-quadruplex in a template strand during transcription. Bull Chem Soc Jpn (2019) 92(3):572–7. doi: 10.1246/bcsj.20180298

[23] Zhang W Li S Li C Li T Huang Y. Remodeling tumor microenvironment with natural products to overcome drug resistance. Front Immunol (2022) 13:1051998. doi: 10.3389/fimmu.2022.1051998 36439106 PMC9685561

[24] Kosiol N Juranek S Brossart P Heine A Paeschke K. G-Quadruplexes: A promising target for cancer therapy. Mol Cancer (2021) 20(1):40. doi: 10.1186/s12943-021-01328-4 33632214 PMC7905668

[25] Carvalho J Mergny JL Salgado GF Queiroz JA Cruz C. G-Quadruplex, friend or foe: The role of the G-quartet in anticancer strategies. Trends Mol Med (2020) 26(9):848–61. doi: 10.1016/j.molmed.2020.05.002 32467069

[26] Abiri A Lavigne M Rezaei M Nikzad S Zare P Mergny JL. Unlocking G-quadruplexes as antiviral targets. Pharmacol Rev (2021) 73(3):897–923. doi: 10.1124/pharmrev.120.000230 34045305

[27] Ruggiero E Richter SN. G-Quadruplexes and G-quadruplex ligands: Targets and tools in antiviral therapy. Nucleic Acids Res (2018) 46(7):3270–83. doi: 10.1093/nar/gky187 PMC5909458 29554280

[28] Wang YH Yang QF Lin X Chen D Wang ZY Chen B. G4ldb 2.2: A database for discovering and studying G-quadruplex and I-motif ligands. Nucleic Acids Res (2022) 50(D1):D150–d60. doi: 10.1093/nar/gkab952 PMC8728129 34718746

[29] Hilton J Gelmon K Bedard PL Tu D Xu H Tinker AV. Results of the phase I cctg Ind.231 trial of cx-5461 in patients with advanced solid tumors enriched for DNA-repair deficiencies. Nat Commun (2022) 13(1):3607. doi: 10.1038/s41467-022-31199-2 35750695 PMC9232501

[30] Sundquist WI Heaphy S. Evidence for interstrand quadruplex formation in the dimerization of human immunodeficiency virus 1 genomic rna. Proc Natl Acad Sci USA (1993) 90(8):3393–7. doi: 10.1073/pnas.90.8.3393 PMC46306 8475087

[31] Ruggiero E Zanin I Terreri M Richter SN. G-Quadruplex targeting in the fight against viruses: An update. Int J Mol Sci (2021) 22(20):10984. doi: 10.3390/ijms222010984 34681641 PMC8538215

[32] De Nicola B Lech CJ Heddi B Regmi S Frasson I Perrone R. Structure and possible function of a G-quadruplex in the long terminal repeat of the proviral hiv-1 genome. Nucleic Acids Res (2016) 44(13):6442–51. doi: 10.1093/nar/gkw432 PMC5291261 27298260

[33] Amrane S Kerkour A Bedrat A Vialet B Andreola M-L Mergny J-L. Topology of a DNA G-quadruplex structure formed in the hiv-1 promoter: A potential target for anti-hiv drug development. J Am Chem Soc (2014) 136(14):5249–52. doi: 10.1021/ja501500c 24649937

[34] Perrone R Nadai M Frasson I Poe JA Butovskaya E Smithgall TE. A dynamic G-quadruplex region regulates the hiv-1 long terminal repeat promoter. J Medicinal Chem (2013) 56(16):6521–30. doi: 10.1021/jm400914r PMC3791109 23865750

[35] Butovskaya E Heddi B Bakalar B Richter SN Phan AT. Major G-quadruplex form of hiv-1 ltr reveals a (3+1) folding topology containing a stem-loop. J Am Chem Soc (2018) 140(42):13654–62. doi: 10.1021/jacs.8b05332 PMC6202629 30299955

[36] Perrone R Nadai M Poe JA Frasson I Palumbo M Palu G. Formation of a unique cluster of G-quadruplex structures in the hiv-1 nef coding region: Implications for antiviral activity. PloS One (2013) 8(8):e73121. doi: 10.1371/journal.pone.0073121 24015290 PMC3754912

[37] Piekna-Przybylska D Sullivan MA Sharma G Bambara RA. U3 region in the hiv-1 genome adopts a G-quadruplex structure in its rna and DNA sequence. Biochemistry (2014) 53(16):2581–93. doi: 10.1021/bi4016692 PMC4007979 24735378

[38] Wang SR Min YQ Wang JQ Liu CX Fu BS Wu F. A highly conserved G-rich consensus sequence in hepatitis c virus core gene represents a new anti-hepatitis c target. Sci Adv (2016) 2(4):e1501535. doi: 10.1126/sciadv.1501535 27051880 PMC4820367

[39] Bian WX Xie Y Wang XN Xu GH Fu BS Li S. Binding of cellular nucleolin with the viral core rna G-quadruplex structure suppresses hcv replication. Nucleic Acids Res (2019) 47(1):56–68. doi: 10.1093/nar/gky1177 30462330 PMC6326805

[40] Fleming AM Ding Y Alenko A Burrows CJ. Zika virus genomic rna possesses conserved G-quadruplexes characteristic of the flaviviridae family. ACS Infect Dis (2016) 2(10):674–81. doi: 10.1021/acsinfecdis.6b00109 PMC5067700 27737553

[41] Majee P Pattnaik A Sahoo BR Shankar U Pattnaik AK Kumar A. Inhibition of zika virus replication by G-Quadruplex-Binding ligands. Mol Therapy-Nucleic Acids (2021) 23:691–701. doi: 10.1016/j.omtn.2020.12.030 PMC7851496 33575115

[42] Belmonte-Reche E Serrano-Chacon I Gonzalez C Gallo J Banobre-Lopez M. Potential G-quadruplexes and I-motifs in the sars-Cov-2. PloS One (2021) 16(6):e0250654. doi: 10.1371/journal.pone.0250654 34101725 PMC8186786

[43] Ji DY Juhas M Tsang CM Kwok CK Li YS Zhang Y. Discovery of G-Quadruplex-Forming sequences in sars-Cov-2. Briefings Bioinf (2021) 22(2):1150–60. doi: 10.1093/bib/bbaa114 PMC7314185 32484220

[44] Bezzi G Piga EJ Binolfi A Armas P. Cnbp binds and unfolds in vitro G-quadruplexes formed in the sars-Cov-2 positive and negative genome strands. Int J Mol Sci (2021) 22(5):2614. doi: 10.3390/ijms22052614 33807682 PMC7961906

[45] Artusi S Ruggiero E Nadai M Tosoni B Perrone R Ferino A. Antiviral activity of the G-quadruplex ligand Tmpyp4 against herpes simplex virus-1. Viruses-Basel (2021) 13(2):196. doi: 10.3390/v13020196 PMC7911665 33525505

[46] Artusi S Nadai M Perrone R Biasolo MA Palu G Flamand L. The herpes simplex virus-1 genome contains multiple clusters of repeated G-quadruplex: Implications for the antiviral activity of a G-quadruplex ligand. Antiviral Res (2015) 118:123–31. doi: 10.1016/j.antiviral.2015.03.016 PMC7113899 25843424

[47] Kumar S Ramamurthy C Choudhary D Sekar A Patra A Bhavesh NS. Contrasting roles for G-quadruplexes in regulating human bcl-2 and virus homologues kshv ks-Bcl-2 and ebv Bhrf1. Sci Rep (2022) 12(1):5019. doi: 10.1038/s41598-022-08161-9 35322051 PMC8943185

[48] Zheng AJL Thermou A Gallardo PG Malbert-Colas L Daskalogianni C Vaudiau N. The different activities of rna G-quadruplex structures are controlled by flanking sequences. Life Sci Alliance (2022) 5(2):e202101232. doi: 10.26508/lsa.202101232 34785537 PMC8605322

[49] Murat P Zhong J Lekieffre L Cowieson NP Clancy JL Preiss T. G-Quadruplexes regulate Epstein-Barr virus-encoded nuclear antigen 1 mrna translation. Nat Chem Biol (2014) 10(5):358–U64. doi: 10.1038/nchembio.1479 24633353 PMC4188979

[50] Kumar S Choudhary D Patra A Bhavesh NS Vivekanandan P. Analysis of G-quadruplexes upstream of herpesvirus mirnas: Evidence of G-quadruplex mediated regulation of kshv mir-K12-1-9,11 cluster and hcmv mir-Us33. BMC Mol Cell Biol (2020) 21(1):67. doi: 10.1186/s12860-020-00306-w 32972365 PMC7513282

[51] Ravichandran S Kim YE Bansal V Ghosh A Hur J Subramani VK. Genome-wide analysis of regulatory G-quadruplexes affecting gene expression in human cytomegalovirus. PloS Pathog (2018) 14(9):e1007334. doi: 10.1371/journal.ppat.1007334 30265731 PMC6179306

[52] Madireddy A Purushothaman P Loosbroock CP Robertson ES Schildkraut CL Verma SC. G-Quadruplex-Interacting compounds alter latent DNA replication and episomal persistence of kshv. Nucleic Acids Res (2016) 44(8):3675–94. doi: 10.1093/nar/gkw038 PMC4856979 26837574

[53] Carvalho J Lopes-Nunes J Campello MPC Paulo A Milici J Meyers C. Human papillomavirus G-rich regions as potential antiviral drug targets. Nucleic Acid Ther (2021) 31(1):68–81. doi: 10.1089/nat.2020.0869 33121376

[54] Biswas B Kandpal M Vivekanandan P. A G-quadruplex motif in an envelope gene promoter regulates transcription and virion secretion in hbv genotype b. Nucleic Acids Res (2017) 45(19):11268–80. doi: 10.1093/nar/gkx823 PMC5737607 28981800

[55] Meier-Stephenson V Badmalia MD Mrozowich T Lau KCK Schultz SK Gemmill DL. Identification and characterization of a G-quadruplex structure in the pre-core promoter region of hepatitis b virus covalently closed circular DNA. J Biol Chem (2021) 296:100589. doi: 10.1016/j.jbc.2021.100589 33774051 PMC8094906

[56] Molnar OR Vegh A Somkuti J Smeller L. Characterization of a G-quadruplex from hepatitis b virus and its stabilization by binding Tmpyp4, Braco19 and Phendc3. Sci Rep (2021) 11(1):23243. doi: 10.1038/s41598-021-02689-y 34853392 PMC8636512

[57] Lavezzo E Berselli M Frasson I Perrone R Palu G Brazzale AR. G-Quadruplex forming sequences in the genome of all known human viruses: A comprehensive guide. PloS Comput Biol (2018) 14(12):e1006675. doi: 10.1371/journal.pcbi.1006675 30543627 PMC6307822

[58] Perrone R Butovskaya E Daelemans D Palu G Pannecouque C Richter SN. Anti-Hiv-1 activity of the G-quadruplex ligand braco-19. J Antimicrob Chemother (2014) 69(12):3248–58. doi: 10.1093/jac/dku280 25103489

[59] Tosoni E Frasson I Scalabrin M Perrone R Butovskaya E Nadai M. Nucleolin stabilizes G-quadruplex structures folded by the ltr promoter and silences hiv-1 viral transcription. Nucleic Acids Res (2015) 43(18):8884–97. doi: 10.1093/nar/gkv897 PMC4605322 26354862

[60] Butovskaya E Solda P Scalabrin M Nadai M Richter SN. Hiv-1 nucleocapsid protein unfolds stable rna G-quadruplexes in the viral genome and is inhibited by G-quadruplex ligands. ACS Infect Dis (2019) 5(12):2127–35. doi: 10.1021/acsinfecdis.9b00272 PMC6909241 31646863

[61] Scalabrin M Frasson I Ruggiero E Perrone R Tosoni E Lago S. The cellular protein hnrnp A2/B1 enhances hiv-1 transcription by unfolding ltr promoter G-quadruplexes. Sci Rep (2017) 7:45244. doi: 10.1038/srep45244 28338097 PMC5364415

[62] Perrone R Doria F Butovskaya E Frasson I Botti S Scalabrin M. Synthesis, binding and antiviral properties of potent core-extended naphthalene diimides targeting the hiv-1 long terminal repeat promoter G-quadruplexes. J Medicinal Chem (2015) 58(24):9639–52. doi: 10.1021/acs.jmedchem.5b01283 PMC4690987 26599611

[63] Belachew B Gao J Byrd AK Raney KD. Hepatitis c virus nonstructural protein Ns3 unfolds viral G-quadruplex rna structures. J Biol Chem (2022) 298(11):102486. doi: 10.1016/j.jbc.2022.102486 36108740 PMC9582721

[64] Jaubert C Bedrat A Bartolucci L Di Primo C Ventura M Mergny JL. Rna synthesis is modulated by G-quadruplex formation in hepatitis c virus negative rna strand. Sci Rep (2018) 8:8120. doi: 10.1038/s41598-018-26582-3 29802381 PMC5970142

[65] Goertz GP Abbo SR Fros JJ Pijlman GP. Functional rna during zika virus infection. Virus Res (2018) 254:41–53. doi: 10.1016/j.virusres.2017.08.015 28864425

[66] Zou M Li JY Zhang MJ Li JH Huang JT You PD. G-Quadruplex binder pyridostatin as an effective multi-target zikv inhibitor. Int J Biol Macromol (2021) 190:178–88. doi: 10.1016/j.ijbiomac.2021.08.121 34461156

[67] Oliva R Mukherjee S Manisegaran M Campanile M Del Vecchio P Petraccone L. Binding properties of rna quadruplex of sars-Cov-2 to berberine compared to telomeric DNA quadruplex. Int J Mol Sci (2022) 23(10):5690. doi: 10.3390/ijms23105690 35628500 PMC9145931

[68] Moraca F Marzano S D'Amico F Lupia A Di Fonzo S Vertecchi E. Ligand-based drug repurposing strategy identified sars-Cov-2 rna G-quadruplex binders. Chem Commun (2022) 58(85):11913–6. doi: 10.1039/d2cc03135c 36196950

[69] Zhai LY Su AM Liu JF Zhao JJ Xi XG Hou XM. Recent advances in applying G-quadruplex for sars-Cov-2 targeting and diagnosis: A review. Int J Biol Macromol (2022) 221:1476–90. doi: 10.1016/j.ijbiomac.2022.09.152 PMC9482720 36130641

[70] Zhang R Xiao K Gu Y Liu H Sun X. Whole genome identification of potential G-quadruplexes and analysis of the G-quadruplex binding domain for sars-Cov-2. Front Genet (2020) 11:587829. doi: 10.3389/fgene.2020.587829 33329730 PMC7728997

[71] Panera N Tozzi AE Alisi A. The G-Quadruplex/Helicase world as a potential antiviral approach against covid-19. Drugs (2020) 80(10):941–6. doi: 10.1007/s40265-020-01321-z PMC7246970 32451923

[72] Maiti AK. Identification of G-quadruplex DNA sequences in sars-Cov2. Immunogenetics (2022) 74(5):455–63. doi: 10.1007/s00251-022-01257-6 PMC8931451 35303126

[73] Qin G Zhao CAQ Liu Y Zhang C Yang G Yang J. Rna G-quadruplex formed in sars-Cov-2 used for covid-19 treatment in animal models. Cell Discov (2022) 8(1):86. doi: 10.1038/s41421-022-00450-x 36068208 PMC9447362

[74] Qin SS Chen XL Xu ZC Li T Zhao SH Hu R. Telomere G-triplex lights up thioflavin T for rna detection: New wine in an old bottle. Anal Bioanal Chem (2022) 414(20):6149–56. doi: 10.1007/s00216-022-04180-7 PMC9208972 35725832

[75] Frasson I Nadai M Richter SN. Conserved G-quadruplexes regulate the immediate early promoters of human alphaherpesviruses. Molecules (2019) 24(13):2375. doi: 10.3390/molecules24132375 31252527 PMC6651000

[76] Artusi S Perrone R Lago S Raffa P Di Iorio E Palu G. Visualization of DNA G-quadruplexes in herpes simplex virus 1-infected cells. Nucleic Acids Res (2016) 44(21):10343–53. doi: 10.1093/nar/gkw968 PMC5137459 27794039

[77] Callegaro S Perrone R Scalabrin M Doria F Palu G Richter SN. A core extended naphtalene diimide G-quadruplex ligand potently inhibits herpes simplex virus 1 replication. Sci Rep (2017) 7:2341. doi: 10.1038/s41598-017-02667-3 28539620 PMC5443766

[78] Saranathan N Biswas B Patra A Vivekanandan P. G-Quadruplexes may determine the landscape of recombination in hsv-1. BMC Genomics (2019) 20:382. doi: 10.1186/s12864-019-5731-0 31096907 PMC6524338

[79] Martins RP Findakly S Daskalogianni C Teulade-Fichou MP Blondel M Fahraeus R. In cellulo protein-mrna interaction assay to determine the action of G-Quadruplex-Binding molecules. Molecules (2018) 23(12):3124. doi: 10.3390/molecules23123124 30501034 PMC6321085

[80] Lista MJ Martins RP Billant O Contesse MA Findakly S Pochard P. Nucleolin directly mediates Epstein-Barr virus immune evasion through binding to G-quadruplexes of Ebna1 mrna. Nat Commun (2017) 8:16043. doi: 10.1038/ncomms16043 28685753 PMC5504353

[81] Reznichenko O Quillevere A Martins RP Loaec N Kang H Lista MJ. Novel cationic Bis(Acylhydrazones) as modulators of Epstein-Barr virus immune evasion acting through disruption of interaction between nucleolin and G-quadruplexes of Ebna1 mrna. Eur J Medicinal Chem (2019) 178:13–29. doi: 10.1016/j.ejmech.2019.05.042 31173968

[82] Westdorp KN Terhune SS. Impact of rna polymerase I inhibitor cx-5461 on viral kinase-dependent and -independent cytomegalovirus replication. Antiviral Res (2018) 153:33–8. doi: 10.1016/j.antiviral.2018.02.014 PMC6015744 29458130

[83] Dabral P Babu J Zareie A Verma SC. Lana And hnrnp A1 regulate the translation of Lana mrna through G-quadruplexes. J Virol (2020) 94(3):e01508-19. doi: 10.1128/jvi.01508-19 31723020 PMC7000962

[84] Metifiot M Amrane S Litvak S Andreola ML. G-Quadruplexes in viruses: Function and potential therapeutic applications. Nucleic Acids Res (2014) 42(20):12352–66. doi: 10.1093/nar/gku999 PMC4227801 25332402

[85] Marusic M Hosnjak L Krafcikova P Poljak M Viglasky V Plavec J. The effect of single nucleotide polymorphisms in G-rich regions of high-risk human papillomaviruses on structural diversity of DNA. Biochim Et Biophys Acta-General Subj (2017) 1861(5):1229–36. doi: 10.1016/j.bbagen.2016.11.007 27836759

[86] Tluckova K Marusic M Tothova P Bauer L Sket P Plavec J. Human papillomavirus G-quadruplexes. Biochemistry (2013) 52(41):7207–16. doi: 10.1021/bi400897g 24044463

[87] Krafcikova P Demkovicova E Halaganova A Viglasky V. Putative hiv and siv G-quadruplex sequences in coding and noncoding regions can form G-quadruplexes. J Nucleic Acids (2017) 2017:6513720. doi: 10.1155/2017/6513720 29464116 PMC5804116

[88] Buket O Clement L DanZhou Y. DNA G-Quadruplex and its potential as anticancer drug target. Sci China Chem (2014) 57(12):1605–14. doi: 10.1007/s11426-014-5235-3 PMC4863707 27182219

[89] Norseen J Johnson FB Lieberman PM. Role for G-quadruplex rna binding by Epstein-Barr virus nuclear antigen 1 in DNA replication and metaphase chromosome attachment. J Virol (2009) 83(20):10336–46. doi: 10.1128/jvi.00747-09 PMC2753104 19656898

[90] Tao YF Zheng YG Zhai QQ Wei DG. Recent advances in the development of small molecules targeting rna G-quadruplexes for drug discovery. Bioorg Chem (2021) 110:104804. doi: 10.1016/j.bioorg.2021.104804 33740677

[91] Shu H Zhang R Xiao K Yang J Sun X. G-Quadruplex-Binding proteins: Promising targets for drug design. Biomolecules (2022) 12(5):648. doi: 10.3390/biom12050648 35625576 PMC9138358

[92] Tateishi-Karimata H Muraoka T Kinbara K Sugimoto N. G-Quadruplexes with Tetra(Ethylene glycol)-modified deoxythymidines are resistant to nucleases and inhibit hiv-1 reverse transcriptase. ChemBioChem (2016) 17(15):1399–402. doi: 10.1002/cbic.201600162 27251574

[93] Seto W-K Lo Y-R Pawlotsky J-M Yuen M-F. Chronic hepatitis b virus infection. Lancet (2018) 392(10161):2313–24. doi: 10.1016/s0140-6736(18)31865-8 30496122

[94] Hayes CN Chayama K. Interferon stimulated genes and innate immune activation following infection with hepatitis b and c viruses. J Med Virol (2017) 89(3):388–96. doi: 10.1002/jmv.24659 27509053

[95] Schneider WM Chevillotte MD Rice CM. Interferon-stimulated genes: A complex web of host defenses. Annu Rev Immunol (2014) 32:513–45. doi: 10.1146/annurev-immunol-032713-120231 PMC4313732 24555472

[96] Li X GG Lu M Chai W Li Y Tong X Li JJX. Long noncoding rna lnc-mxa inhibits beta interferon transcription by forming rna-DNA triplexes at its promoter. J Virol (2019) 93(21):e00786–19. doi: 10.1007/s00281-017-0636-y PMC6803265 31434735

[97] Hirashima K Seimiya H. Telomeric repeat-containing Rna/G-Quadruplex-Forming sequences cause genome-wide alteration of gene expression in human cancer cells in vivo. Nucleic Acids Res (2015) 43(4):2022–32. doi: 10.1093/nar/gkv063 PMC4344506 25653161

[98] Matsumoto K Okamoto K Okabe S Fujii R Ueda K Ohashi K. G-Quadruplex-Forming nucleic acids interact with splicing factor 3b subunit 2 and suppress innate immune gene expression. Genes Cells (2021) 26(2):65–82. doi: 10.1111/gtc.12824 33290632 PMC7898707

[99] Sun J Wu G Pastor F Rahman N Wang WH Zhang Z. Rna helicase Ddx5 enables Stat1 mrna translation and interferon signalling in hepatitis b virus replicating hepatocytes. Gut (2022) 71(5):991–1005. doi: 10.1136/gutjnl-2020-323126 34021034 PMC8606016

[100] Kwok CK Marsico G Sahakyan AB Chambers VS Balasubramanian S. Rg4-seq reveals widespread formation of G-quadruplex structures in the human transcriptome. Nat Methods (2016) 13:841–44. doi: 10.1038/nmeth.3965 27571552

[101] Chan KL Peng B Umar MI Chan CY Sahakyan AB Le MTN. Structural analysis reveals the formation and role of rna G-quadruplex structures in human mature micrornas. Chem Commun (Camb) (2018) 54(77):10878–81. doi: 10.1039/c8cc04635b 30204160

[102] Pandey S Agarwala P Jayaraj GG Gargallo R Maiti S. The rna stem-loop to G-quadruplex equilibrium controls mature microrna production inside the cell. Biochemistry (2015) 54(48):7067–78. doi: 10.1021/acs.biochem.5b00574 26554903

[103] Ghosh A Pandey SP Ansari AH Sundar JS Singh P Khan Y. Alternative splicing modulation mediated by G-quadruplex structures in Malat1 lncrna. Nucleic Acids Res (2022) 50(1):378–96. doi: 10.1093/nar/gkab1066 PMC8754661 34761272

[104] Zhang H Chen X Zhang J Wang X Chen H Liu L. Long noncoding rnas in hbvrelated hepatocellular carcinoma (Review). Int J Oncol (2020) 56(1):18–32. doi: 10.3892/ijo.2019.4909 31746420

[105] Lei B Song H Xu F Wei Q Wang F Tan G. When does hepatitis b virus meet long-stranded noncoding rnas? Front Microbiol (2022) 13:962186. doi: 10.3389/fmicb.2022.962186 36118202 PMC9479684

[106] Loureiro D Tout I Narguet S Benazzouz SM Mansouri A Asselah T. Mirnas as potential biomarkers for viral hepatitis b and c. Viruses (2020) 12(12):1440. doi: 10.3390/v12121440 33327640 PMC7765125

[107] Xie KL Zhang YG Liu J Zeng Y Wu H. Micrornas associated with hbv infection and hbv-related hcc. Theranostics (2014) 4(12):1176–92. doi: 10.7150/thno.8715 PMC4183996 25285167

[108] O'Day E Le MTN Imai S Tan SM Kirchner R Arthanari H. An rna-binding protein, Lin28, recognizes and remodels G-quartets in the micrornas (Mirnas) and mrnas it regulates. J Biol Chem (2015) 290(29):17909–22. doi: 10.1074/jbc.M115.665521 PMC4505039 26045559

[109] Yuan JH Tu JL Liu GC Chen XC Huang ZS Chen SB. Visualization of ligand-induced c-myc duplex-quadruplex transition and direct exploration of the altered c-myc DNA-protein interactions in cells. Nucleic Acids Res (2022) 50(8):4246–57. doi: 10.1093/nar/gkac245 PMC9071431 35412611

[110] Pagano A Iaccarino N Abdelhamid MAS Brancaccio D Garzarella EU Di Porzio A. Common G-quadruplex binding agents found to interact with I-Motif-Forming DNA: Unexpected multi-Target-Directed compounds. Front Chem (2018) 6:281. doi: 10.3389/fchem.2018.00281 30137743 PMC6066642

[111] De Rache A Mergny J-L. Assessment of selectivity of G-quadruplex ligands *Via* an optimised fret melting assay. Biochimie (2015) 115:194–202. doi: 10.1016/j.biochi.2015.06.002 26079222

[112] Asamitsu S Bando T Sugiyama H. Ligand design to acquire specificity to intended G-quadruplex structures. Chem – A Eur J (2019) 25(2):417–30. doi: 10.1002/chem.201802691 30051593

